# C/EBPβ deletion in oncogenic Ras skin tumors is a synthetic lethal event

**DOI:** 10.1038/s41419-018-1103-y

**Published:** 2018-10-15

**Authors:** Zachary J. Messenger, Jonathan R. Hall, Dereje D. Jima, John S. House, Hann W. Tam, Debra A. Tokarz, Robert C. Smart

**Affiliations:** 1 Toxicology Program, Raleigh, NC USA; 2 Center of Human Health and the Environment, Raleigh, NC USA; 3 Department of Biological Sciences, Raleigh, NC USA; 4 Bioinformatics Research Center, Raleigh, NC USA; 5 Department of Population Health and Pathobiology, North Carolina State University, Raleigh, NC USA

## Abstract

Therapeutic targeting of specific genetic changes in cancer has proven to be an effective therapy and the concept of synthetic lethality has emerged. CCAAT/enhancer-binding protein-β (C/EBPβ), a basic leucine zipper transcription factor, has important roles in cellular processes including differentiation, inflammation, survival, and energy metabolism. Using a genetically engineered mouse model, we report that the deletion C/EBPβ in pre-existing oncogenic Ha-Ras mouse skin tumors in vivo resulted in rapid tumor regression. Regressing tumors exhibited elevated levels of apoptosis and p53 protein/activity, while adjacent C/EBPβ-deleted skin did not. These results indicate that the deletion of C/EBPβ de-represses p53 in oncogenic Ras tumors but not in normal wild-type Ras keratinocytes, and that C/EBPβ is essential for survival of oncogenic Ras tumors. Co-deletion of C/EBPβ and p53 in oncogenic Ras tumors showed p53 is required for tumor regression and elevated apoptosis. In tumors, loss of a pathway that confers adaptability to a stress phenotype of cancer/tumorigenesis, such as DNA damage, could result in selective tumor cell killing. Our results show that oncogenic Ras tumors display a significant DNA damage/replicative stress phenotype and these tumors have acquired a dependence on C/EBPβ for their survival. RNAseq data analysis of regressing tumors deleted of C/EBPβ indicates a novel interface between p53, type-1 interferon response, and death receptor pathways, which function in concert to produce activation of extrinsic apoptosis pathways. In summary, the deletion of C/EBPβ in oncogenic Ras skin tumors is a synthetic lethal event, making it a promising target for future potential anticancer therapies.

## Introduction

Ras proteins are small GTPase membrane-bound signal transducers and the coding genes are one of the most frequently mutated dominant oncogenic drivers in human cancer^1–3^. At least one of the three family members (Ha, N, or Ki-RAS) is mutated in 20–30% of all human cancers, with some cancers exhibiting high percentages of Ras mutation; e.g., > 90% of pancreatic cancers contain mutated Ki-RAS^1^. Once mutated, oncogenic Ras signaling promotes proliferation through activation of the RAF-MAPK, PI3K, and RAL-GDS pathways^4–9^. These pathways also downregulate pro-apoptotic genes, leading to conditions that favor expansion while evading cell death^10^.

CCAAT/enhancer binding protein-β (C/EBPβ), a basic leucine zipper transcription factor, has important roles in cellular processes including differentiation, inflammation, survival, and energy metabolism^11–13^. C/EBPβ is activated by numerous cytokines^14–16^, as well as by oncogenic Ras, RTKs, and Toll-like receptors^17–21^. C/EBPβ contributes to cell survival in response to DNA damage, toxicants, or oncogenic stress^22–25^. C/EBPβ levels are increased in numerous human cancers and often are associated with poor prognoses and invasive growth^26–35^. In numerous cancer cell types, C/EBPβ has a prosurvival function^18,22,24,25,32,36^.

In order for tumor cells to acquire the hallmark traits of cancer^37^, which includes the evasion of apoptosis, tumor cells must respond to and overcome the cellular stresses associated with tumorigenesis^38^. These stresses are often referred to as the “stress phenotypes of tumorigenesis/cancer” and include DNA damage, DNA replicative stress, mitotic stress, metabolic stress, proteotoxic stress, and oxidative stress^38^. Loss of a pathway(s) that confers adaptability to stress phenotypes of tumorigenesis could result in selective tumor cell killing^38^. These ideas form the conceptual framework of synthetic lethality, where targeting a specific pathway results in death of tumor cells but has no effect in normal cells^39–42^.

Our results demonstrate that deletion of C/EBPβ in oncogenic Ras-driven skin tumors is a synthetic lethal event. C/EBPβ-depleted tumors displayed activation of a type-1 interferon (IFN) response and de-repression p53 activity to induce death receptor/tumor necrosis factor receptor (TNFR)-mediated apoptosis and tumor regression.

## Materials and methods

### Animal care, treatment/doses, tumor measuring

All animal husbandry, care, and experimentation was conducted per National Institute of Health (NIH) guidelines and approved by the North Carolina State University (NCSU) Institutional Animal Care and Use Committee (IACUC). All mice were backcrossed onto a B6.129 background for at least five generations. K14-CreER^tam^ mice (from Jackson Laboratory Tg(KRT14-cre/ERT)20EFu/J)^43^ were crossed with C/EBPβ^flox/flox^ mice^44^ and p53^flox/flox^ mice (from NCI Mouse Repository FVB.129-Trp53^tm1Brn^)^45^ to obtain the following genotypes, which have been maintained on a B6.129 hybrid background: K14-CreER^tam^ (Cre), K14-CreER^tam^;C/EBPβ^flox/flox^ (IKOβ), K14-CreER^tam^;p53^flox/flox^ (IKOp53), and K14-CreER^tam^;C/EBPβ^flox/flox^;p53^flox/flox^ (DIKO). To induce tumors, mice aged 8–12 weeks had dorsal hair clipped with electric clippers and were given a single topical dose of 200 nmol 7,12-dimethylbenz[a]anthracene (DMBA) (0.2 ml) (Sigma, D3254, St. Louis, MO, USA) in acetone followed 1 week later by thrice weekly dosing of 5 nmol 12-*O*-tetradecanoylphorbol-13-acetate (TPA) (0.2 ml) (Cayman Chemical, 10008014, Ann Arbor, MI, USA) in acetone for the entirety of tumor studies. Activation of the K14-CreER^tam^ was accomplished via dosing 2.5 mg (6.73 μmol) of tamoxifen (Sigma, T5648, St. Louis MO, USA) dissolved in corn oil with 5% ethanol intraperitoneal (i.p.) injected daily (0.25 ml), 5 days/week for 2 weeks. Tumor numbers were tabulated weekly with tumor volume being calculated by measuring height, width, and length of tumors (height × width × length). In vivo 5-Bromo-2’deoxyuridine (BrdU) labeling was carried out as previously described^22^ by i.p. injecting BrdU (Sigma, B5002, St. Louis, MO, USA) in phosphate-buffered saline (PBS, pH 7.4) 1 h before killing at a dose of 100 mg BrdU per kg body mass (typically ~ 0.3 ml).

### DNA sequencing

Whole tumor DNA was collected and Sanger sequencing was performed by the North Carolina State University Genomic Sciences Laboratory (Raleigh, NC, USA). The 61st codon of Ha-Ras was amplified using forward primer 5′-ACTCCTACCGGAAACAGGT-3′ and reverse primer: 5′-GAGGACATCCATCAGTACAG-3′, and sequenced using the reverse primer.

### Preparation of epidermal lysates for SDS-polyacrylamide gel electrophoresis

Mice were killed by cervical dislocation and clipped dorsal skin was removed. To remove epidermis, the skin was submerged in 60 °C dH_2_O for 6 s followed by 10 s in ice water, the skin was dried and the epidermis was scraped off of the dermis and placed in RIPA buffer (1% NP-40, 0.5% sodium deoxycholate, 0.1% SDS, 1 mM dithiothreitol, 1 mM sodium orthovanadate, 1 mM 4-(2-Aminoethyl)benzenesulfonyl fluoride hydrochloride (AEBSF), and 1 × protease inhibitor cocktail (Roche, Indianapolis, IN, USA) in PBS. Collected samples were sonicated on ice and centrifuged at 14,000 × *g* for 10 min. Equal amounts of protein were resolved via SDS-polyacrylamide gel electrophoresis, transferred to polyvinylidene difluoride membrane and probed using the following antibodies: C/EBPβ (Santa Cruz, sc-150, 1:5000, Dallas, TX, USA), α-Tubulin (Santa Cruz, sc-8035, 1:8000, Dallas, TX, USA), and p53 (Cell Signaling, 2524, 1:5000, Danvers, MA, USA).

### Quantification of apoptosis and inflammatory cell infiltration

Shaved mouse dorsal skin was fixed in 10% neutral buffered formalin for 24 h, changed to 70% ethanol, and embedded in paraffin, or tissues were fixed in PAX gene tissue fixative (PreAnalytiX, 765312, Hombrechtikon, Switzerland) for 24 h, changed to PAXgene stabilizer (PreAnalytiX, 765512, Hombrechtikon, Switzerland), and embedded in paraffin. Tissue sections (5 μM) were stained with hematoxylin and eosin. Apoptotic cells were scored as positive if they met all three following criteria: (1) dark pyknotic nuclei, (2) cytoplasmic eosinophilia, and (3) detachment from adjacent cells^18,22,23^. Apoptotic positive cells were expressed as positive cells per mm^2^ area of parenchyna. Area was measured using ImageJ software^46^. Apoptosis in the normal epidermis adjacent to tumors was scored in the interfollicular epidermis as previously described above and expressed as positive cells per cm skin. Apoptosis was confirmed with cleaved caspase 3 (Cell Signaling, 9661, 1:1000, Danvers, MA, USA) immunohistochemical (IHC) and Terminal deoxynucleotidyl transferase dUTP nick end labeling (TUNEL) (Promega, G3250, Madison, WI, USA) staining using UVB-treated mouse skin as a positive control and untreated mouse skin used as negative control. Inflammation was scored based on infiltrating neutrophils and mononuclear leukocytes, which were scored separately for intratumoral and extratumoral skin using a scoring system adapted from a method previously described^47^ on a scale of 0–3, where 0: none present, 1: few present, 2: moderate occurrence, and 3: abundant occurrence. Intratumoral included the epidermal components of the papillomas with the subjacent dermis and subcutis. All slides were scored by D. Tokarz (board certified veterinary pathologist from CHHE Comparative Pathology Core), who was blinded as to treatment group, with the exception of slides labeled as controls. Slides were scored in a random order.

### Tumor histological analysis

Using hematoxylin and eosin (H&E)-stained 5 µm sections, skin lesions were identified and scored by a veterinary pathologist and scored as follows: (1) Papilloma: a discrete mass > 1 mm in depth that displayed hyperplasia with mild dysplasia. (2) Carcinoma in situ: a discrete raised hyperplasia that is > 1 mm in depth with expanded dermis. The dysplasia includes the loss of transepidermal differentiation, increased in mitotic index, and increased in nuclear to cytoplasm ratio. (3) Microinvasive squamous cell carcinoma: in addition to the criteria in carcinoma in situ, the lesion has increase depth in dermis with expansion through basement membrane. (4) Squamous cell carcinoma: squamous cell carcinoma that touches or penetrates the muscle layer. Categories (3) and (4) are considered as malignant skin tumors.

### Immunohistochemical staining

Clipped mouse dorsal skin was fixed in 10% neutral buffered formalin for 24 h, changed to 70% ethanol, and embedded in paraffin, or tissues were fixed in PAXgene tissue fixative for 24 h, changed to PAXgene stabilizer, and embedded in paraffin. Tissue sections (5 μm) were deparaffinized, peroxidases were inactivated with 3% H_2_O_2_, and subjected to antigen retrieval using a 2100 retriever (Aptum, Southampton, UK) with citrate buffer (pH 6). Next, sections were treated with 3% H_2_O_2_ once more before being blocked with normal goat serum (or normal horse serum) before incubation overnight at 4 °C with one of the following antibodies: C/EBPβ (Santa Cruz, sc-150, 1:4000, Dallas, TX, USA with C/EBPβ^−/−^ mouse skin used as negative control and wild-type mouse skin used as positive control, p53 (Cell Signaling, 2524, 1:1000, Danvers, MA, USA) with p53^−/−^ mouse skin used as negative control and UVB-treated mouse skin used as positive control, Keratin 5 (Covance, PRB-160P, 1:2000, Princeton, NJ, USA) with wild-type mouse skin used as positive control, Keratin 10 (Covance, PRB159P, 1:2000, Princeton, NJ, USA) with wild-type mouse skin used as positive control, Ki67 (Bethyl Labs, IHC-00375, 1:500, Montgomery, TX, USA) with wild-type mouse skin used as positive control, γH2AX (Bethyl Labs, A300-081A, 1:2000, Montgomery, TX, USA) and phospho-p53 (Ser15) (murine serine 18 (S18)) (Cell Signaling, 9284, 1:1000, Danvers, MA, USA) with skin from a gamma radiation dosed mouse as a positive control and wild-type mouse skin as a negative control, and cleaved caspase 8 using wild-type mouse skin as a negative control. (Novus Biologicals, 56116, 1:2000, Littleton, CO). Staining was visualized using species appropriate secondary antibodies from Vectastain Elite ABC kits (Vector Labs, mouse:PK-6102 rabbit:PK-6101, Burlingame CA, USA) and DAB Peroxidase Substrate Kit (Vector Labs, SK-4100, Burlingame, CA, USA). Sections were counterstained with hematoxylin and quantification was calculated as positive cells per mm^2^ area of parenchyma. Area was measured using ImageJ software^46^. Positively stained cells in the normal epidermis adjacent to tumors were scored in the interfollicular epidermis and expressed as positive cells per cm skin. Immunohistochemical staining for BrdU was carried out as previously described^22^ by deparaffinizing 5 μm sections and incubating in the following, 2 M HCl for 30 min at 37 °C, boric acid-borate buffer for 3 min at room temperature, 0.05 M Tris-HCl (pH 7.8 in 0.1% CaCl_2_) with 0.01% trypsin for 3 min at 37 °C, and 3% H_2_O_2_ for 10 min at room temperature. Sections were blocked in normal horse serum for 30 min and then incubated for 1 h at room temperature with anti-BrdU IgG primary antibody (BD Biosciences, 69138, 1:100, San Jose, CA, USA). Staining was visualized and quantified as described above. All IHC staining was performed with listed controls as well as no primary antibody controls. All controls behaved as expected. Immunohistochemical analysis for Mouse CD4 (eBioscience, 14-9766, 1:25, Waltham, MA, USA), Mouse CD8a (eBioscience, 14-0808,1:100, Waltham, MA, USA), and Mouse F4/80 (AbD Serotec, Cat # MCA497RT, 1:25, Hercules, CA, USA) was performed by the Animal Histopathology and Lab Medicine Core located at the University of North Carolina School of Medicine (Chapel Hill, NC, USA).

### RNA isolation and next-generation sequencing

Total RNA was extracted from whole tumors collected from Cre and IKOβ mice following cessation of tamoxifen treatment as described above (week 21 of TPA) and were homogenized in Qiazol (Qiagen, 79306, Hilden, Germany). RNA extraction was carried out using the Quick-RNA MiniPrep Kit (Zymo Research, 11-328, Irvine, CA, USA). Total RNA samples were submitted to the North Carolina State University Genomic Sciences Laboratory for Illumina RNA library construction and sequencing. Sample integrity and concentration were evaluated using an RNA 6000 Nano Chip on the Agilent Bioanalyzer 2100 (Agilent, Santa Clara, CA, USA). Purification of messenger RNA (mRNA) was performed using the oligo-dT beads provided in the NEBNext Poly(A) mRNA Magnetic Isolation Module (New England Biolabs, Ipswich, MA, USA). NEBNext Ultra Directional RNA Library Prep Kit (New England Biolabs, Ipswich, MA, USA) and NEBNext Multiplex Oligos for Illumina (New England Biolabs, Ipswich, MA, USA) were used to make the cDNA libraries for Illumina sequencing using the manufacturer-specified protocol, which involved chemically fragmenting the mRNA and priming it with random oligos for first-strand cDNA synthesis. Second-strand cDNA synthesis was then carried out with dUTPs to preserve strand orientation information. The cDNA was purified, end repaired, and “a-tailed” for adaptor ligation. Next, the samples were selected for a final library size of 400–550 bp using sequential AMPure XP bead isolation (Beckman Coulter, Brea, CA, USA). Library enrichment was performed and specific indexes for each sample were added during the protocol-specified PCR amplification. The amplified library fragments were purified and checked for quality and final concentration using an Agilent 2200 Tapestation (Agilent, Santa Clara, CA, USA). The final quantified libraries were sequenced using Illumina’s NextSeq 500 DNA sequencer, utilizing a 75 bp paired end kit (Illumina, San Diego, CA, USA), which gave around 200 million reads for the 8 samples (4 samples/group), which works out to ~25 million reads/sample. The software package Real Time Analysis (RTA) was used to generate raw bcl, or base call files, which were then de-multiplexed by sample into fastq files for data submission.

RNA-Seq data analysis was conducted in consultation with the Bioinformatics Core of the NCSU Center of Human Health and the Environment. The quality of raw sequence data was assessed using FastQC and the first 12 poor-quality bases were trimmed based on the quality matrix from the FastQC, application. The remaining good quality reads were aligned to the mouse reference genome (mm38 version 87) using STAR^48^ aligner. For each replicate sample, per-gene counts of uniquely mapped reads were calculated using *htseq-count* script from the HTSeq python package. Genes with numerous aliases were removed and represented in the data as a single gene. The count matrix was imported and normalized for sequence depth and distortion, and dispersion was estimated using DESeq2^49^ Bioconductor package in the R statistical computing environment. Differentially expressed genes were identified after applying multiple testing correction using the Benjamini–Hockeberg procedure^50^ (false discovery rate (FDR) < 0.1).

### Gene set enrichment analysis

Gene set enrichment analysis (GSEA) is a computational method that determines whether an a priori defined set of genes shows statistically significant, concordant differences between two biological states utilizing GSEA software^51,52^. Briefly, the genes from differential expression were ranked based on signed fold change × − log10 *p*-value. The rank file contains genes with the strongest upregulation (top), strongest downregulation (bottom), and not changing are in the middle. The analysis was preformed using GseaPreranked application in the GSEA software using mouse C2 MSigDB gene set and default settings. Finally, the enriched pathways (FDR < 0.1) were plotted using Enrichment Map Visualization in the same software package and plotted the bar plot for the top 40 enriched pathways.

For visualization of specific pathways, normalized counts from DESeq2^49^ were row scaled (by gene), grouped by average linkage, and heatmaps generated. Data for each ontology along with adjusted *p*-values are provided in Supplemental Tables 3-6. Ontologies were retrieved March 2017 and combined in the following manner from the curated C2.GSEA MSigDB database available at (http://www.software.broadinstitute.org/gsea/msigdb):

**Interferon** – ("INTERFERON-GAMMA SIGNALING PATHWAY%PANTHER PATHWAY%P00035" plus "INTERFERON-GAMMA SIGNALING%REACTOME%R-HSA-877300.1" plus "INTERFERON SIGNALING%REACTOME%R-HSA-913531.1" plus "INTERFERON ALPHA BETA SIGNALING%REACTOME%R-HSA-909733.1" plus 10 additional OAS genes), **p53** – (“HALLMARK_P53_PATHWAY%MSIGDB_C2%HALLMARK_P53_PATHWAY“), **TNF** –(“HALLMARK_TNFA_SIGNALING_VIA_NFKB%MSIGDB_C2%HALLMARK_TNFA_SIGNALING_VIA_NFKB“), and **Death Receptor** – ("BIOCARTA_DEATH_PATHWAY%MSIGDB_C2%BIOCARTA_DEATH_PATHWAY" plus "DEATH RECEPTOR SIGNALLING%REACTOME DATABASE ID RELEASE 59%73887").

## Results

### Spatial and temporal regulation of C/EBPβ in epidermis and pre-existing oncogenic Ras skin tumors

**Fig. 1:**
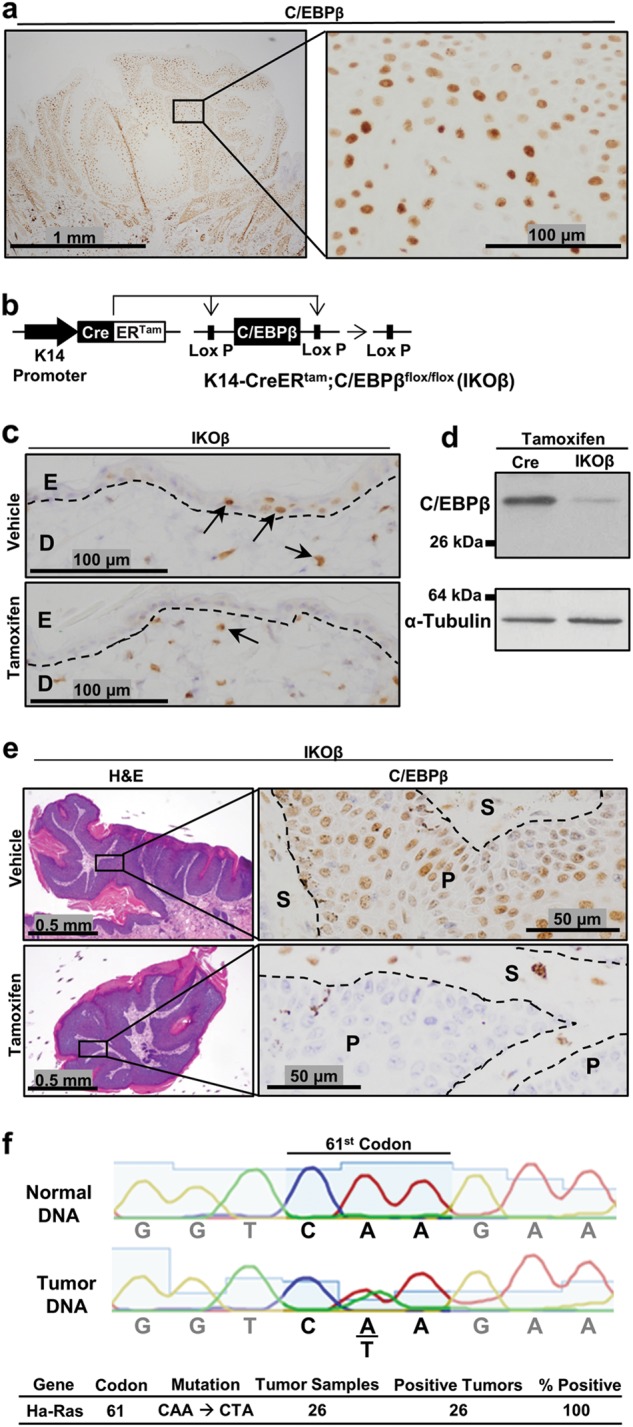
Spatial and temporal regulation of C/EBPβ in epidermis and in pre-existing oncogenic Ras skin tumors. **a** Immunohistochemical (IHC) staining for C/EBPβ in a DMBA/TPA-induced mouse squamous papilloma. **b** Schematic of the K14-CreER^tam^;C/EBPβ^flox/flox^ (IKOβ) transgenic mouse model system. **c** IHC staining for C/EBPβ in IKOβ mouse skin following vehicle or tamoxifen dosing. D dermis, E epidermis. **d** Western blot analysis for C/EBPβ in epidermal lysates from tamoxifen-treated Cre and IKOβ mice. **e** H&E and C/EBPβ IHC staining in DMBA/TPA-induced mouse squamous papilloma following vehicle and tamoxifen dosing. **f** DNA sequence of the 61st codon of Ha-Ras in mouse tail and DMBA/TPA-induced squamous papillomas

C/EBPβ is abundantly expressed throughout the epithelial portion (parenchyma) of oncogenic Ras containing mouse skin tumors (squamous papillomas) arising from the DMBA/TPA protocol (Fig. 1a). To determine whether C/EBPβ is essential for the survival of oncogenic Ha-Ras skin tumors, we developed a mouse model, K14-CreER^tam^;C/EBPβ^flox/flox^ (IKOβ), in which C/EBPβ could be conditionally and temporally deleted in the epidermis and in pre-existing oncogenic Ha-Ras skin tumors (Fig. 1b). As shown in Fig. 1c, dosing IKOβ mice with tamoxifen i.p. (1 ×/day for 5 days/week for 2 weeks) resulted in loss of C/EBPβ protein in the epidermis, whereas dermal C/EBPβ levels were unaffected. Western blot analysis of epidermal lysates confirmed C/EBPβ deletion (Fig. 1d).

To test whether C/EBPβ could be deleted in pre-existing skin tumors in this mouse IKOβ model, we generated skin tumors in IKOβ mice using a DMBA/TPA tumorigenesis treatment protocol^53^. Following development of oncogenic Ras-driven tumors, mice were then treated with tamoxifen or vehicle i.p. and tumors were collected 2 weeks after the start of tamoxifen treatment. Tamoxifen treatment resulted in the loss of C/EBPβ protein in the parenchyma portion of the tumor, but not in the stroma (Fig. 1e). DNA sequencing of DMBA/TPA tumors from K14-CreER^tam^ (Cre) mice confirmed the expected A- > T mutation in the 61st codon of Ha-Ras (Fig. 1f)^54^. Thus, the IKOβ mouse is a tractable model to test whether C/EBPβ is required for survival of oncogenic Ras skin tumors.

### Oncogenic Ras skin tumors depend on C/EBPβ for survival

**Fig. 2:**
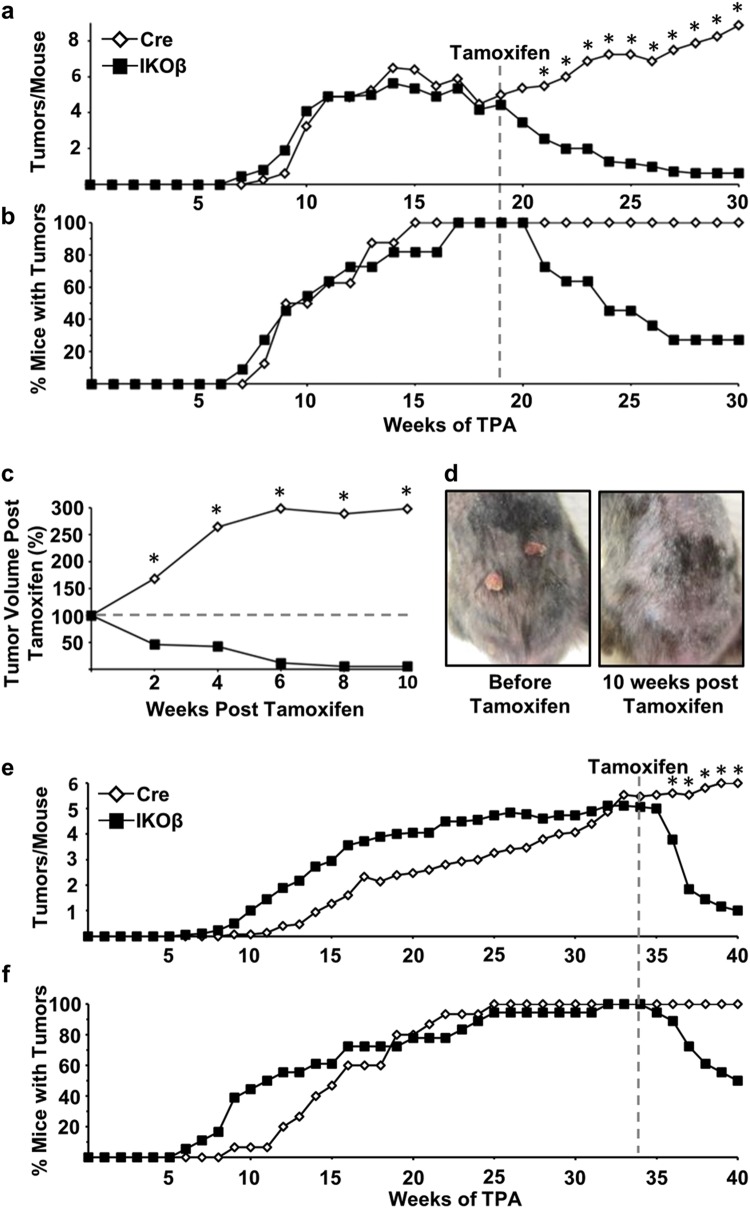
Oncogenic Ras skin tumors depend on C/EBPβ for survival. **a** Tumor multiplicity in Cre and IKOβ before and after mice were dosed with tamoxifen starting at 19 weeks, **b** tumor incidence, and **c** tumor volume remaining (Cre mice *n* = 10, IKOβ mice *n* = 11). **d** Representative photographs of skin tumors before and after tamoxifen on the same mouse. **e** Tumor multiplicity in Cre and IKOβ mice before and after mice were dosed with tamoxifen starting at 34 weeks, and **f** tumor incidence (Cre mice *n* = 15, IKOβ mice *n* = 18). Data are expressed as means. *indicates significantly different from IKOβ controls *p* < 0.05 via the Student’s *t*-test

Cre and IKOβ mice were initiated with DMBA and promoted with TPA for 30 weeks. By week 19 of TPA treatment, 100% of mice from both genotypes developed an average of ~5 tumors/mouse. At week 19, mice were treated with tamoxifen i.p. (1 ×/day for 5 days/week for 2 weeks). At 10 weeks after the start of tamoxifen treatment, ~80% of the IKOβ tumors had completely regressed (Fig. 2a) and the percentage of mice with tumors decreased to ~25% (Fig. 2b). Using tumor volume at 19 weeks as a standard, tumor volume in Cre mice tripled, whereas it decreased by 98% in IKOβ mice after 10 weeks of tamoxifen treatment (Fig. 2c). A representative IKOβ mouse before and after tamoxifen treatment is shown in Fig. 2d. At the termination of this experiment (30 weeks TPA treatment), skin tumors from Cre mice were collected and histopathological analysis showed that 78% of skin tumors were squamous papillomas and 22% were squamous cell carcinomas.

To determine whether more progressed skin tumors also regress upon the C/EBPβ deletion, we conducted another DMBA/TPA tumor study where mice were treated with tamoxifen at 34 weeks of TPA treatment (compared with 19 weeks in the previous experiment). Six weeks after initiation of tamoxifen treatment, ~80% of IKOβ mouse tumors completely regressed (Fig. 2e) and the percentage of mice with tumors decreased to 50% (Fig. 2f). This tumor experiment was terminated at 40 weeks, as some tumor bearing mice developed a cachexia-like phenotype. In summary, these results demonstrate that deletion of C/EBPβ in oncogenic Ras tumors results in tumor regression.

### Regressing tumors display elevated levels of apoptosis and p53 protein while adjacent C/EBPβ-depleted skin does not

**Fig. 3:**
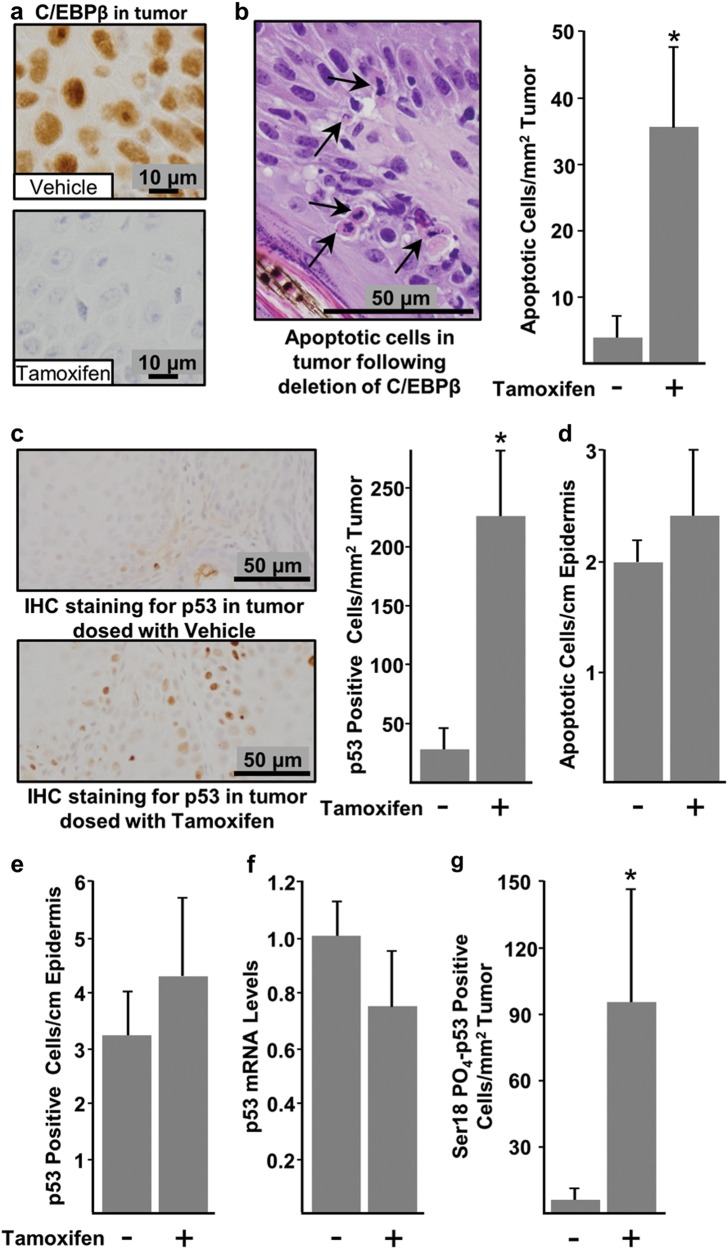
Regressing C/EBPβ-deficient tumors display tumor-specific elevations in apoptosis and p53 protein, whereas adjacent C/EBPβ-depleted skin is unaffected. Tumor bearing IKOβ mice were dosed with either vehicle control or tamoxifen at 19 weeks and tumors were collected 2 weeks later. **a** Deletion of C/EBPβ was confirmed by IHC staining. **b** Photograph displaying apoptotic cells (left) and quantification of apoptosis in H&E-stained tumors (right) (vehicle *n* = 18 tumors, tamoxifen *n* = 10 tumors). **c** Photograph displaying p53 IHC staining (left) and quantification of IHC staining for p53 (right) (vehicle *n* = 15 tumors, tamoxifen *n* = 12 tumors). **d** Quantification of apoptosis in H&E-stained adjacent normal epidermis (vehicle *n* = 5 mice, tamoxifen *n* = 8 mice). **e** Quantification of IHC staining for p53 in adjacent normal epidermis (vehicle *n* = 5 mice, tamoxifen *n* = 8 mice). **f** Quantification of p53 mRNA from tumors (vehicle *n* = 3 tumors, tamoxifen *n* = 3 tumor). **g** Quantification of IHC staining for p53 phosphorylated on serine 18 (vehicle *n* = 9 tumors, tamoxifen *n* = 11 tumors). Data are expressed as mean ± SD. *indicates significantly different from controls *p* < 0.05 via the Student’s *t*-test

To gain insight into the mechanism of tumor regression, another group of IKOβ mice were initiated with DMBA, promoted with TPA, and at 19 weeks of TPA treatment, mice were dosed with either tamoxifen or vehicle. Tumors, along with adjacent skin, were collected 2 weeks after the start tamoxifen or vehicle treatment, and PAXgene-fixed paraffin-embedded (PFPE) sections were prepared. The deletion of C/EBPβ in regressing tumors and adjacent skin was confirmed by lack of IHC staining for C/EBPβ protein (Fig. 3a). Based on the characteristic morphology of apoptotic cells in H&E-stained sections (Fig. 3b), we observed a ~7-fold increase in apoptotic cells in the parenchyma of regressing tumors (C/EBPβ deleted) compared with the parenchyma of non-regressing tumors (C/EBPβ^+/+^) (Fig. 3b). Both TUNEL and cleaved caspase 3 staining produced similar numbers of apoptotic cells as observed in the H&E sections (data not shown). The increase in apoptosis in the regressing tumors was accompanied by a ~9-fold increase in p53-positive tumor cells (Fig. 3c). Unlike tumors, there were no significant differences in apoptosis or p53 levels in epidermis adjacent to the tumor when C/EBPβ was depleted or not (Fig. 3d, e). Moreover, the levels of apoptosis and p53 were low in the adjacent normal epidermis, regardless of C/EBPβ status (Fig. 3d, e). These results demonstrate that the loss of C/EBPβ leads to increased levels of p53 and apoptotic cell death in oncogenic Ras skin tumor cells but not in adjacent epidermal keratinocytes with wild-type Ras, indicating the loss of C/EBPβ is a synthetic lethal event in oncogenic Ras tumors. We observed that the increase in p53 protein levels in regressing tumors was not accompanied by increased p53 mRNA levels (Fig. 3f) but was accompanied by increased phosphorylation of S18 of p53 (human serine 15) (Fig. 3g). S18 phosphorylation of p53 is mediated by Ataxia-Telangiesctasia Mutated (ATM)/Ataxia Telangiectasia And Rad3-Related Protein (ATR) in response to DNA damage^55,56^ and contributes to p53 stabilization and enhances p53-dependent transactivation^57^.

### Skin tumors exhibit increased levels of DNA damage, a stress phenotype of tumorigenesis

**Fig. 4:**
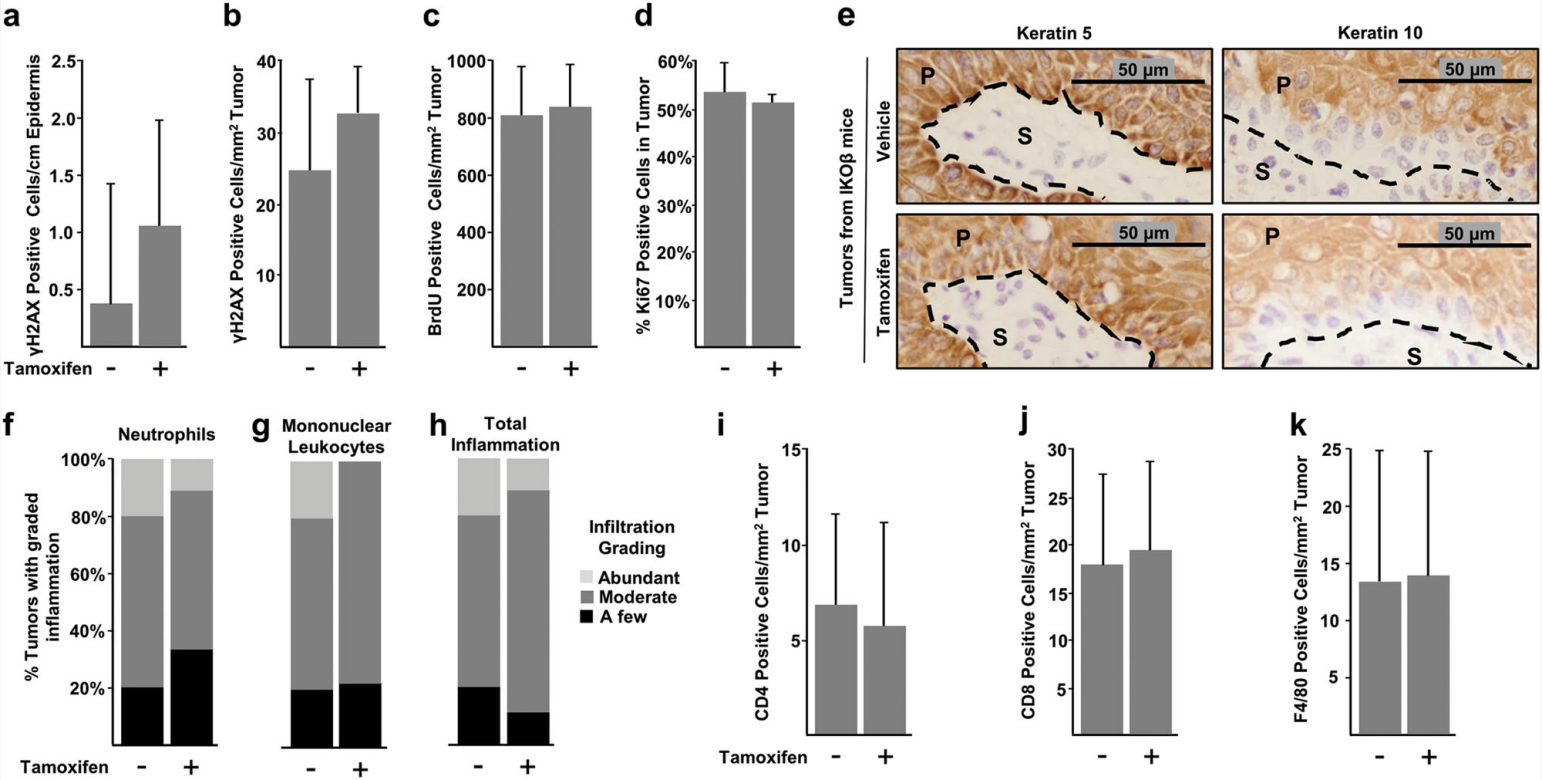
Skin tumors exhibit DNA damage and regressing C/EBPβ-deficient tumors do not display differences in proliferation, senescence, differentiation or inflammation. Tumor bearing IKOβ mice were dosed with either vehicle control or tamoxifen and tumors were collected two weeks after the initial vehicle or tamoxifen dose. **a** Quantification of IHC staining for Phospho-Histone H2A.X (γH2AX) in adjacent normal epidermis (vehicle *n* = 4 mice, tamoxifen *n* = 4 mice). **b** Quantification of γH2AX in tumor (vehicle *n* = 13 tumors, tamoxifen *n* = 8 tumors). **c** Quantification of IHC staining for BrdU incorporation in tumors (vehicle *n* = 17 tumors, tamoxifen *n* = 9 tumors). **d** Quantification of IHC staining for Ki67 in tumors (vehicle *n* = 13 tumors, tamoxifen *n* = 9 tumors). **e** Photograph of IHC staining for Keratin 5 (left) and Keratin 10 (right). Quantification of **f** neutrophil infiltration, **g** mononuclear leukocyte infiltration, and **h** total inflammation (vehicle *n* = 18 tumors, tamoxifen *n* = 18 tumors) from H&E-stained tumor sections. **i** Quantification of CD4 IHC staining in tumor parenchyma and stroma (vehicle *n* = 7 tumors, tamoxifen *n* = 7 tumors). **j** Quantification of CD8 IHC staining in tumor parenchyma and stroma (vehicle *n* = 10 tumors, tamoxifen *n* = 9 tumors). **k** Quantification of F4/80 IHC staining in tumor parenchyma and stroma (vehicle *n* = 11 tumors, tamoxifen *n* = 9 tumors). Data are expressed as mean ± SD or percent counts. *indicates significantly different from controls *p* < 0.05 via the Student’s *t*-test

Prior work in our lab has demonstrated that the epidermal C/EBPβ-knockout mice treated with DNA-damaging agents display increased levels of p53 and apoptosis compared with similarly treated wild-type mice^18,22,23^. In the current study, the presence of DNA damage and subsequent stress phenotypes in oncogenic Ras skin tumors could provide a mechanism whereby loss of C/EBPβ results in increased sensitivity to DNA damage, leading to increased p53 levels and apoptosis. To test for DNA damage, we measured γH2AX, a marker of double-strand breaks^58^ in both tumors and adjacent epidermis. We observed very low numbers of γH2AX-positive cells in the adjacent epidermis of regressing and non-regressing tumors (Fig. 4a). However, γH2AX IHC positive cells were significantly and similarly increased in both regressing and non-regressing tumors (Fig. 4b). These results indicate that regressing and non-regressing skin tumors display a hallmark tumor stress phenotype involving DNA damage stress^38^. However, only regressing tumors depleted of C/EBPβ display increased p53 (Fig. 3c), phospho-p53(S18) (Fig. 3g), and apoptosis (Fig. 3b), suggesting the activation of p53 in these Ha-Ras regressing tumors occurs via enhancement of pathways that are activated by endogenous DNA damage stress as a result C/EBPβ depletion.

### Regressing C/EBPβ-deficient tumors do not display differences in proliferation, senescence, differentiation, or inflammation

Examination of regressing and non-regressing tumors for BrdU, Ki67, and keratin 5 and 10 showed no difference in proliferation, senescence, or differentiation, respectively (Fig. 4c–e). Further, tumor regression was not accompanied by a significantly different inflammatory response as measured by neutrophil or mononuclear leukocyte infiltration (Fig. 4f–h). IHC staining for CD4, CD8, and F4/80 antigens indicative of CD4+ T-cells, CD8+ T-cells, and macrophages respectively, revealed no statistically significant differences between regressing and non-regressing tumors as well (Fig. 4i–k). In summary, of the cellular processes evaluated, only increased apoptosis and increased p53 levels are associated with the regressing tumor phenotype.

### Development of a mouse model for the inducible conditional co-deletion of C/EBPβ and p53 in epidermis and in pre-existing oncogenic Ras skin tumors

**Fig. 5:**
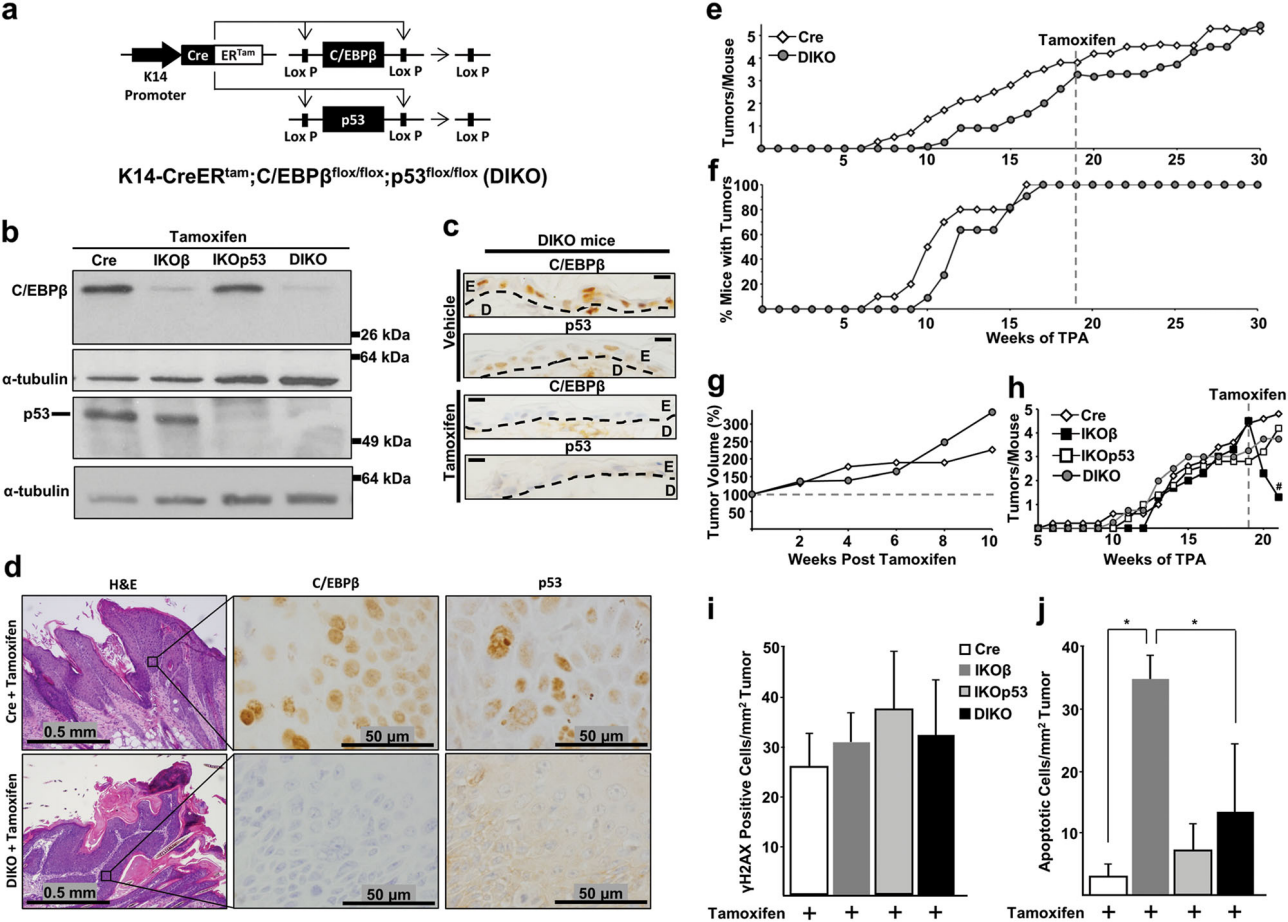
Oncogenic Ras tumor regression following deletion of C/EBPβ is dependent on p53. **a** Schematic of the K14-CreER^tam^;C/EBPβ^flox/flox^;p53^flox/flox^ (DIKO) transgenic mouse model system. **b** Western blot analysis of C/EBPβ and p53 in epidermal lysates of tamoxifen-treated Cre, IKOβ, IKOp53, and DIKO mice. **c** IHC staining for C/EBPβ and p53 in normal DIKO mouse skin following vehicle and tamoxifen treatment. D dermis, E epidermis. Scale bar: 10 μm. **d** H&E and IHC staining for C/EBPβ and p53 in DMBA/TPA-induced mouse squamous papillomas following tamoxifen dosing. **e** Tumor multiplicity in DMBA/TPA-induced skin squamous papilloma in Cre and DIKO mice dosed with tamoxifen at 19 weeks of skin tumor promotion (Cre *N* = 10 mice, DIKO *N* = 11 mice). **f** Tumor incidence in Cre and DIKO mice. **g** Tumor volume in Cre and DIKO mice. **h** Tumor multiplicity in Cre, IKOβ, IKOp53, and DIKO mice before and after tamoxifen collected 2 weeks after initial tamoxifen dose (Cre *N* = 5 mice, IKOβ *N* = 3 mice, IKOp53 *N* = 5 mice, DIKO *N* = 5 mice). # indicates IKOβ is significantly different from Cre *p* < 0.05 via the Student’s *t*-test. **i** Quantification of γH2AX IHC staining (Cre *n* = 9 tumors, IKOβ *n* = 3 tumors, IKOp53 *n* = 7 tumors, DIKO *n* = 7 tumors). **j** Quantification of apoptosis in H&E-stained tumors collected 2 weeks after the initial tamoxifen dose (Cre *n* = 9 tumors, IKOβ *n* = 3 tumors, IKOp53 *n* = 7 tumors, DIKO *n* = 7 tumors). Data are expressed as mean ± SD. *Significantly different from controls *p* < 0.05 via the Student’s *t*-test

To determine whether p53 is required for the observed increase in apoptosis and tumor regression following deletion of C/EBPβ, we developed an inducible conditional C/EBPβ-p53 double knockout mouse K14-CreER^tam^;C/EBPβ^flox/flox^;p53^flox/flox^ (DIKO) (Fig. 5a) in which C/EBPβ and p53 could be spatially and temporally co-deleted in the epidermis and in pre-existing oncogenic Ha-Ras skin tumors. Dosing DIKO mice with tamoxifen resulted in the loss of both C/EBPβ and p53 protein in the epidermis (Fig. 5b, c). To test whether C/EBPβ and p53 could be co-deleted in pre-existing skin tumors, we generated skin tumors in Cre and DIKO mice using a DMBA/TPA tumorigenesis treatment protocol and treated with tamoxifen. Following tamoxifen treatment, both C/EBPβ and p53 protein in the epithelial (parenchyma) portion of the DIKO skin tumors were deleted (Fig. 5d).

### Oncogenic Ras skin tumors are dependent on C/EBPβ for survival and deletion of C/EBPβ in these tumors is a synthetic lethal event dependent upon p53

Cre and DIKO mice were initiated with DMBA, promoted with TPA for 30 weeks, and treated with tamoxifen beginning at week 19 of TPA treatment. No tumor regression was observed in the tamoxifen-treated DIKO and these mice, and similar to the Cre mice, continued to develop additional tumors (Fig. 5e, f). Cre and DIKO mice displayed an increase in tumor volume after tamoxifen treatment (Fig. 5g) and there was no decrease in the percent mice with tumors in either group (Fig. 5f).

As part of this experiment, a small group of Cre, IKOβ, IKOp53, and DIKO mice were also initiated, promoted, and treated with tamoxifen and tumors from these mice were collected for further analysis 2 weeks after the start of tamoxifen treatment. IKOβ mice displayed tumor regression, whereas Cre, IKOp53, and DIKO mice showed no regression (Fig. 5h) and displayed a similar number of tumors/mouse. PFPE sections of the tumors from Cre, IKOβ, IKOp53, and DIKO mice were analyzed for apoptosis and γH2AX levels. All tumors displayed a tumor stress phenotype involving DNA damage stress as determined by significant of γH2AX-positive cells (Fig. 5i).

Most of the apoptosis observed in the IKOβ mouse tumors was p53 dependent (Fig. 5j). Thus, co-deletion of C/EBPβ and p53 in oncogenic Ras tumors demonstrates that p53 is required for tumor regression and elevated apoptosis.

### Regressing tumors display enrichment of a type I IFN response, p53, and TNF/death receptor signaling networks

**Fig. 6:**
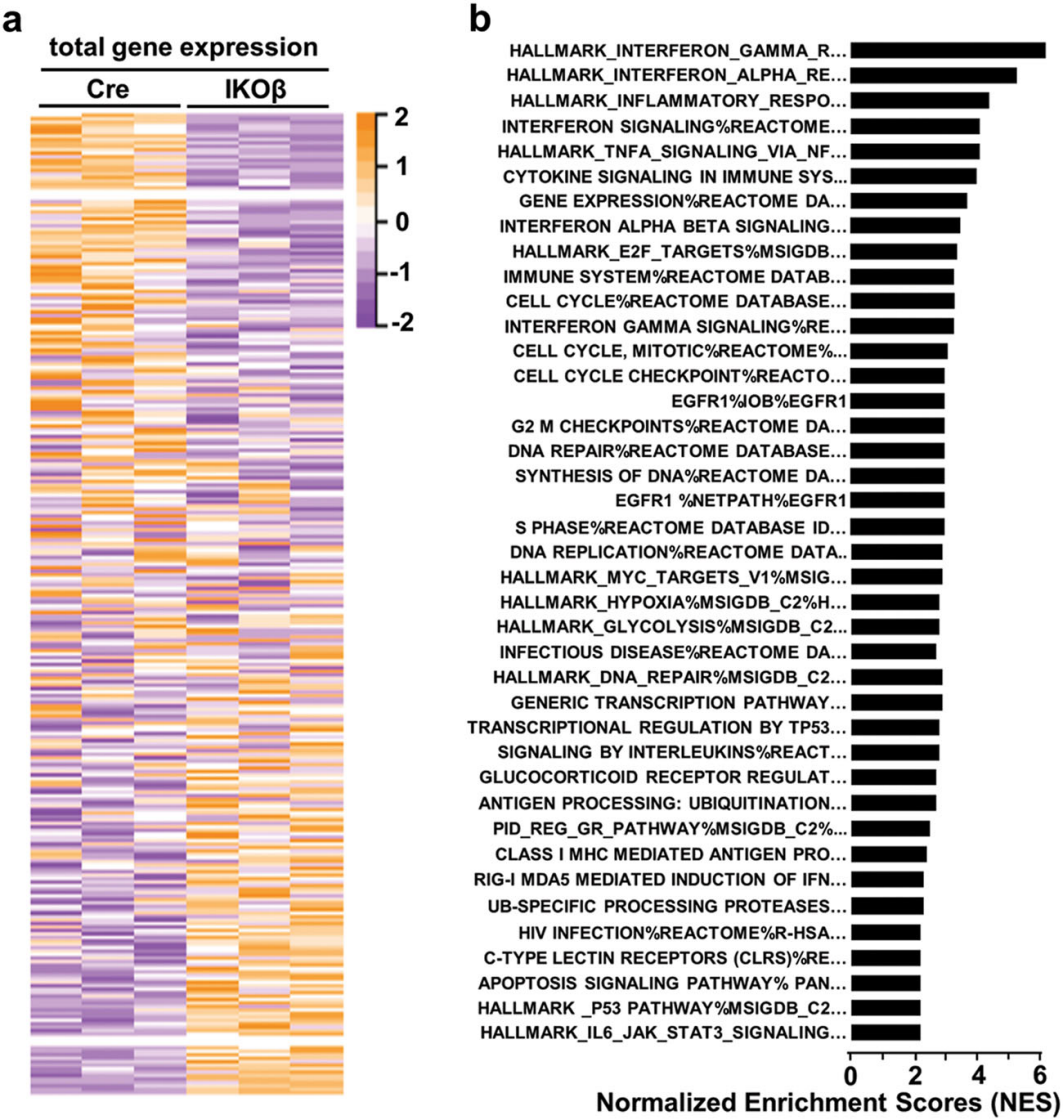
Regressing tumors display significant alterations in gene expression and enrichment of type I interferon response, p53, and TNF/death receptor signaling networks. Total RNA from three Cre and three IKOβ tumors from different mice were subjected to RNA sequencing. **a** Heatmap showing total gene expression. **b** Gene set enrichment analysis with top 40 statistically significant (FDR < 0.1), positively enriched pathways displayed

To further understand the mechanism(s) responsible for synthetic lethality, we conducted RNA sequncing (RNAseq) analysis on RNA isolated from three non-regressing tumors (Cre mice) and three regressing tumors (IKOβ mice) at 2 weeks after the start of tamoxifen treatment. Deletion of C/EBPβ in the tumors had a profound effect on transcriptional responses. We identified a total of 2287 genes (880 upregulated and 1407 downregulated) out of a data set of 18,924 unique genes that were altered in the regressing tumors compared with the non-regressing tumors (FDR < 0.1) (Fig. 6a and Table S1). Expression patterns in the three regressing tumors were similar and distinct from the expression patterns observed in the three non-regressing tumors (Fig. 6a). GSEA was performed using gene sets from within the Molecular Signature Database (MSigDB). Strikingly, GSEA revealed that the IFN pathway was the most highly enriched pathway in the regressing tumors (Fig. 6b, Table S2). GSEA normalized enrichment scores for the TNF pathway, cell cycle checkpoints/DNA repair, and p53 pathway were also among highest in the regressing tumor (FDR < 0.1) (Fig. 6b).

### Deletion of C/EBPβ in oncogenic Ras tumors triggers a subset of ISGs that are unique to the regressing tumors

**Fig. 7:**
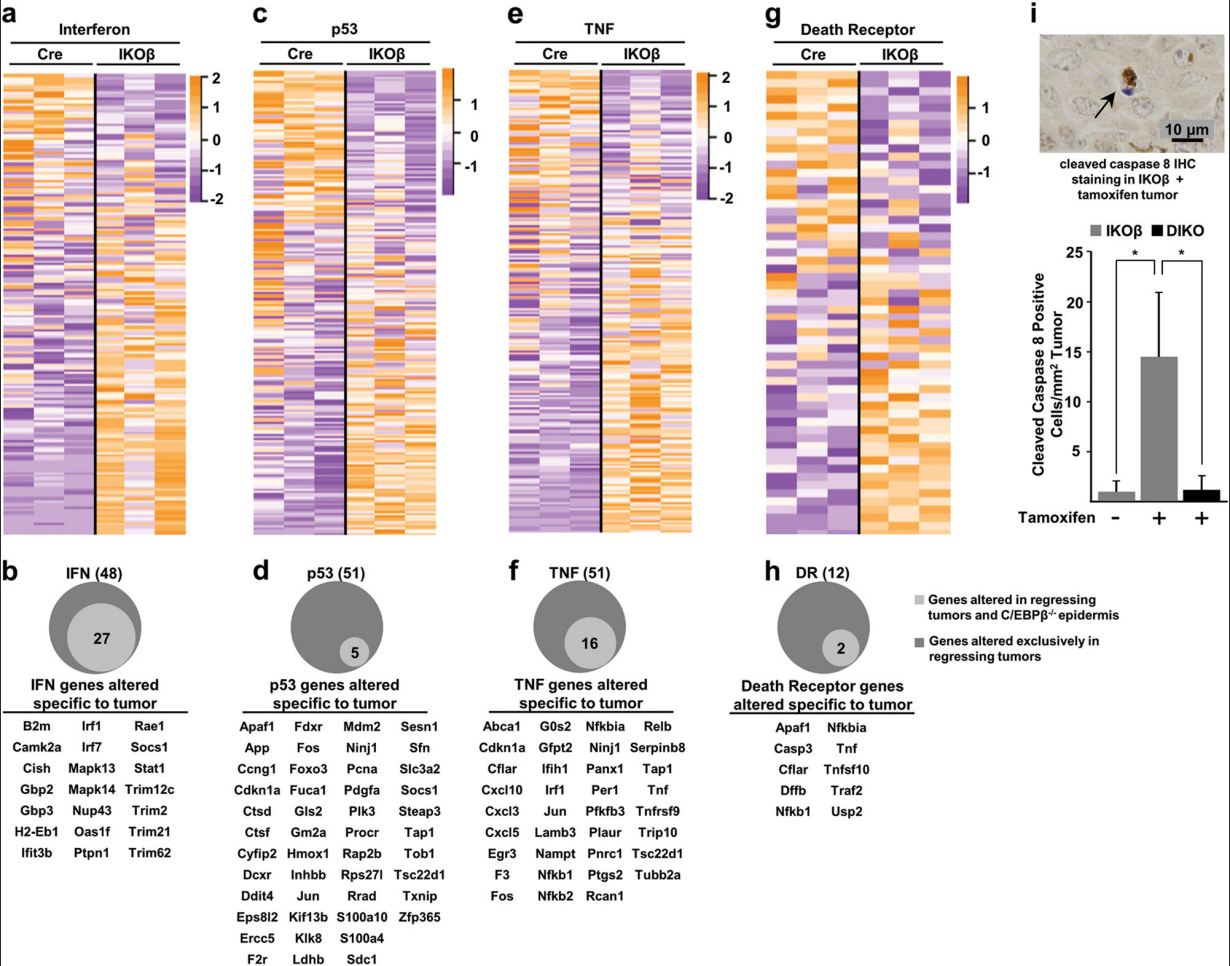
Regressing tumors display enrichment of a type I interferon response, p53, and TNF/death receptor signaling networks. **a** Heatmap of genes in combined MSigDB interferon gene sets. **b** Forty-eight interferon response genes were significantly altered (FDR < 0.1) in C/EBPβ-depleted tumors. The light gray circle represents genes overlapping with C/EBPβ^−/−^ epidermis and the below gene list shows significantly altered genes unique to the regressing tumors, not shared with C/EBPβ^−/−^ epidermis. **c** Heatmap of genes in the MSigDB hallmark p53 pathway gene set. **d** Fifty-one hallmark p53 genes were significantly altered (FDR < 0.1) in C/EBPβ-depleted tumors. The light gray circle represents genes overlapping with C/EBPβ^−/−^ epidermis and the below gene list shows significantly altered genes unique to the regressing tumors, not shared with C/EBPβ^−/−^ epidermis. **e** Heatmap of genes in the MSigDB hallmark TNF signaling gene set. **f** Fifty-one hallmark TNF genes were significantly altered (FDR < 0.1) in C/EBPβ-depleted tumors. The light gray circle represents genes overlapping with C/EBPβ^−/−^ epidermis and the below gene list shows significantly altered genes unique to the regressing tumors, not shared with C/EBPβ^−/−^ epidermis. **g** Heatmap of genes in combined MSigDB death receptor signaling gene sets. **h** Twelve death receptor signaling genes were significantly altered (FDR < 0.1) in C/EBPβ-depleted tumors. The light gray circle represents genes overlapping with C/EBPβ^−/−^ epidermis and the below gene list shows significantly altered genes unique to the regressing tumors, not shared with C/EBPβ^−^^/−^ epidermis. **i** Example photograph of IHC staining for cleaved caspase 8 (above) and quantification of IHC staining for cleaved caspase 8 (below) (vehicle *n* = 5 tumors, IKOβ *n* = 9 tumors, DIKO *n* = 8 tumors). Data are expressed as mean ± SD. *Significantly different from controls *p* < 0.05 via the Student’s *t*-test

Among the 2287 significantly altered genes, it is noteworthy that 16 of the top 50 genes were ISGs (Table S1). Further analysis of the MSigDB combined IFN ontologies showed that 48/161 IFN-stimulated genes (ISGs) were significantly altered in the regressing tumor (FDR < 0.1) (Fig. 7a and Table S3). TaqMAN quantittative reverse-transcriptase PCR analysis for *Ifnβ1* using RNA isolated from 3 regressing and 3 non-regressing tumors revealed that *Ifnβ1* transcripts were not detectable in non-regressing tumors (40 cycles); however, *Ifnβ1* transcripts were increased ~ 100–fold (average Ct 33 cycles) in the regressing tumors. Unlike the deletion of C/EBPβ in oncogenic Ras skin tumors, the deletion of C/EBPβ in mouse epidermis had no effect on apoptosis. To determine whether the 48 ISGs that are significantly altered in the regressing tumor depleted of C/EBPβ are unique to the regressing tumor, we conducted RNAseq analysis on C/EBPβ-deleted epidermis from three K5Cre^+/tg^; C/EBPβ^flox/flox^ and on epidermis from three K5Cre mice. Surprisingly, many ISGs were also significantly altered in the C/EBPβ-deleted epidermis and, of these, 56% overlapped with the 48 ISGs significantly altered in the regressing tumor (Fig. 7b). The 21 ISGs whose expression is unique to the regressing tumor are shown in Fig. 7b, and include *Irf1*, *Irf7*, and *Stat1*.

### Deletion of C/EBPβ results in upregulation of p53 target genes involved in the extrinsic apoptotic pathway

Analysis of MSigDB p53 ontology showed that 51/191 genes associated with the p53 pathway were significantly altered in the regressing tumor (FDR < 0.1) (Fig. 7c, Table S4). Less than 10% of the significantly altered genes associated with p53 pathway in the regressing tumor are significantly altered in C/EBPβ-deleted epidermis further supporting the importance of p53 pathway in tumor regression. The 46 p53 genes whose expression is unique to the regressing tumor and not overlapping with C/EBPβ-deleted epidermis are shown in Fig. 7d. Strikingly, we did not observe altered expression of p53-dependent genes such as *Puma*, *Bax*, and *Noxa* or other genes involved in the intrinsic apoptotic pathway such as *Bim*, *Bad*, *Bid*, and *Bmf* in the regressing tumors. We did observe that *Apaf1* was significantly and selectively upregulated in regressing tumors (FDR < 0.1).

### TNF/death receptor pathways are activated in the regressing tumors

Similar to GSEA, Ingenuity Pathway Analysis (IPA) of regressing and non-regressing tumors also showed that the TNF, IFN, and p53/apoptosis pathways as top canonical pathways altered in the regressing tumors; however, IPA identified the death receptor pathway (22/93 genes) as the number one canonical pathway (*z*-score = 3.8) associated with regressing tumors. Analysis of the gene sets for the TNF and death receptor pathways revealed that 51/195 genes (Fig. 7e and Table S5) and 12/58 genes (Fig. 7g and Table S6), respectively, were significantly altered in the regressing tumors (FDR < 0.1). Of the genes within the TNF and death receptor gene sets that were significantly altered in the regressing tumors, only 31% (16/51) TNF and 17% (2/12) death receptor genes were significantly altered in C/EBPβ-deleted epidermis (Fig. 7f/h). The 35 TNF genes and the 10 death receptor genes whose expression is unique to the regressing tumor and not overlapping with C/EBPβ-deleted epidermis are shown in Fig. 7f/h. Some of these key genes in TNF and death receptor pathways include *Tnf*, *Cflar* (cFLIP), *Traf2*, *Tnfrsf9*, and *Tnfsf10* (TRAIL). Caspase 8 activation (cleaved caspase 8) is a key part of the extrinsic apoptotic pathway and caspase 8 is primarily activated by death receptor and TNFR signaling. To provide evidence that the TNF/death receptor pathways are activated in the regressing tumor, we conducted IHC analysis for cleaved caspase 8 in regressing and non-regressing tumors. IHC analysis revealed regressing tumors displayed a remarkable 14-fold increase in cleaved caspase 8 when compared with non-regressing tumors (Fig. 7i). Moreover, this increase in cleaved caspase 8 was not observed in DIKO tumors.

## Discussion

Previous studies showed that mice lacking C/EBPβ in their epidermis are highly resistant to the development of skin tumors by chemical carcinogens that produce mutations in Ha-Ras^18^. To determine whether C/EBPβ is essential for the survival of oncogenic Ha-Ras tumors, we developed a mouse model where C/EBPβ could be conditionally and temporally deleted in oncogenic Ras tumors. We show that deletion of C/EBPβ in oncogenic Ras tumors resulted in rapid tumor regression that was accompanied by elevated levels of apoptosis and p53. Importantly, this increase in apoptosis and p53 was tumor specific, as neither apoptosis nor p53 levels were increased in C/EBPβ-depleted epidermis adjacent to the tumor. Our results demonstrate that the deletion of C/EBPβ in oncogenic Ras skin tumors is a synthetic lethal event dependent upon p53.

Large-scale synthetic lethal screens using cultured cells and RNA interference have been conducted to identify genes that are required for survival of oncogenic Ras containing cells^39^. In general, these screens have not yielded the positive results initially anticipated and it has been suggested that this is, in part, due to the in vitro screening methods utilized, which do not replicate the selective pressures experienced by tumor cells in vivo^39^. Although our in vivo studies have overcome this in vitro issue, most human tumors display Ki-Ras mutations^1^ and our in vivo mouse skin model exclusively involves Ha-Ras^Q61L^ mutations. Future studies will be required to determine if the synthetic lethal effect of C/EBPβ deletion in vivo in skin tumors is applicable to other mouse model systems/tumor types and forms of oncogenic Ras (Ha, Ki, N).

Previous studies showed C/EBPβ knockout mice treated with DNA-damaging agents display elevated levels of p53 and p53-mediated apoptosis in their epidermis compared with similarly treated wild-type mice^22,23^. These findings indicate that C/EBPβ functions to repress p53 levels/activity and increase cell survival in response to exogenously induced DNA damage. In the current study, we observed elevated levels of DNA damage (based on increased levels of γH2AX) in both regressing and non-regressing oncogenic Ras skin tumors^58^. DNA damage in tumors is known to be induced by oncogenic stress and can occur through stalled replication forks and reactive oxygen species^38,59^. Therefore, we reasoned that the deletion of C/EBPβ may sensitize oncogenic Ras skin tumor cells to the endogenous levels of DNA damage through de-repression of p53 activity. Increased endogenous DNA damage stress is a tumorigenic stress phenotype that tumors are forced to deal with, in order to survive^38,59^ and, in doing so, the tumor may become over reliant on genes such as C/EBPβ to suppress apoptosis downstream of DNA damage. Luo et al.^38^ suggested selective tumor cell killing could be accomplished by stress sensitization or stress overload. Stress sensitization involves blocking a pathway(s) the tumor has evolved to depend on to handle the increased tumorigenic stress, whereas stress overload involves causing additional stress to the tumor resulting in an overload of the already stretched capacity of the tumor to handle this cellular stress. Our results are consistent with the notion that the deletion of C/EBPβ removes a critical stress survival pathway in the tumor and this sensitizes tumor cells to oncogenic stress and tumorigenic stress-induced endogenous DNA damage to stimulate synthetic lethal apoptotic cell death and tumor regression.

**Fig. 8:**
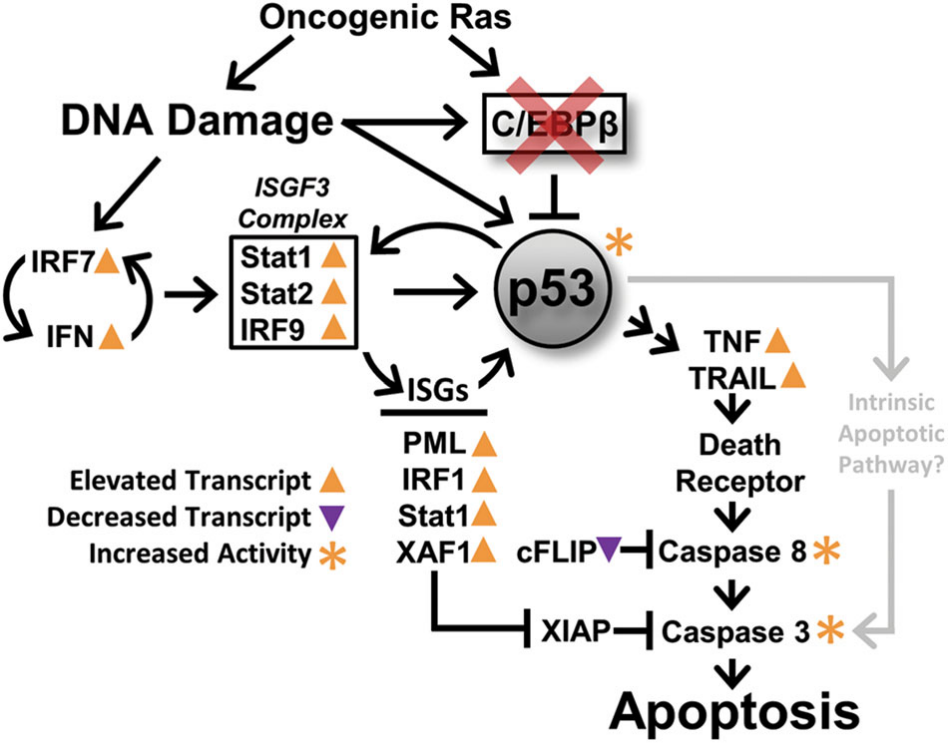
Positive Feedback Working Model. Model depicting the link between oncogenic Ras, DNA damage, type I interferon response, TNF, death receptors, and the p53 pathway to the apoptotic response and tumor regression observed in C/EBPβ-depleted tumors. Arrows and asterixes represent statistically significant up- (orange) or downregulated (purple) transcripts (FDR < 0.1) or proteins (*p* < 0.05) in the C/EBPβ-depleted regressing tumors

Our cleaved caspase 8 results are consistent with the idea that the extrinsic apoptosis pathway involving TNF/TRAIL (TNF-related apoptosis-inducing ligand)/death receptor are activated in the regressing tumor and that p53 is essential for this activation in the C/EBPβ-deleted skin tumors. However, caspase 8 has been reported to be activated by caspase 3 via the intrinsic apoptosis pathways as part of a feedback loop^60^, so we cannot rule out the intrinsic pathways of apoptosis. When comparing regressing vs non-regressing tumors we observed no differences in the expression of key genes involved in p53-dependent intrinsic apoptosis such as *Noxa* and *Puma*^61^ or genes involved in Endoplasmic Reticulum (ER) stress-induced apoptosis such as *Bim*, *Perk*, *Ire1*, and *Atf6*^62,63^. Although no differences in *Puma*, *Noxa*, and *Bim* mRNA levels were detected, it is conceivable that altered posttranslational regulation in the regressing tumors results in increased protein levels of these key genes. It is also possible that the existing levels of Puma, Noxa, and Bim in the regressing tumor are sufficient to cooperate/synergize with the observed upregulation of the extrinsic apoptosis pathway to regulate cell death. Although DNA damage can induce apoptosis through a p53-dependent intrinsic pathway independent of Fas or the death receptor adapter FADD^64^, it has also been reported that TRAIL can mediate p53-dependent apoptosis through the death receptor DR5 in response to DNA damage^65,66^. Our unbiased approaches involving RNAseq analysis strongly point to activation of extrinsic apoptosis pathways exclusively in regressing tumors. Based on our phenotypic results and key genes significantly altered (FDR < 0.1) in the regressing tumors we have developed a working model (Fig. 8), whereby we link oncogenic Ras, DNA damage, type I IFN response, TNF, TRAIL, death receptors, and p53 pathway to the apoptotic response and tumor regression observed in C/EBPβ-depleted tumors. In this model, we propose the deletion of C/EBPβ de-represses p53 and sensitizes tumor cells to the DNA damage induced by oncogenic Ras/tumor stress, and this coupled with an induced type-1 IFN response further activates the de-repressed p53 producing a positive feedback loop, which initiates extrinsic apoptosis signaling and tumor regression through the TNFR and death receptor pathways^67,68^.

IRF7 is activated by pattern-recognition receptors that recognize nucleic acids^69^ and is significantly increased in the regressing tumor. IRF7 is a potent transcriptional regulator of many ISGs including of IFNα/β^69^, and in our model, we propose this results in a continuous stimulation of type-1 IFN response. The expression of numerous key significantly altered ISGs in our GSEA data sets are also regulated by p53 and several ISGs positively regulate p53^70^. For example, several of the upregulated ISGs in the regressing tumors such as *Irf9*, *Stat1*, *Stat2*, *Tnf*, *TRAIL*, *Isg15*, *Xaf1*, *Pml*, and *Mx1* (Tables S3, S4)^65,70–74^ can be regulated by IFN or p53. IRF9, STAT1, and STAT2 form the canonical heterotrimeric ISG factor 3 protein complex, which regulates the transcription of a multitude of ISGs including p53^71,75^. Some of the above mentioned ISGs, including STAT1, PML, IRF1, ISG15, and XAF1 can positively regulate p53 protein activity/stability^71–73,76,77^. This results in a positive feedback loop involving ISGs and de-repressed p53 initiates apoptosis through the TNFR, TRAIL, and death receptor pathways^67,68^. The ISG *Xaf1* is significantly upregulated in the regressing tumors, and XAF1 and TRAIL are major mediators of the extrinsic cell death pathway where XAF1 significantly increases the cellular sensitivity to the pro-apoptotic actions of TRAIL^78^. We also observed a significant downregulation of *Cflar* (cFLIP) a master anti-apoptotic regulator of TNF- and TRAIL-induced apoptosis^68^.

Although p53 is most appreciated for its role in DNA damage and oncogenic stress, it is now evident that p53 has important roles in the immune system, including roles in innate immunity where it enhances type-1 IFN activity^70^. Our results indicate that loss of C/EBPβ in oncogenic Ras tumor cells unleashes a hyperactive type-1 IFN and reveals a novel interface between p53, innate immune response, and death receptor pathways that function together produce tumor regression. Targeting of C/EBPβ could hold promise as future potential cancer therapy.

## Electronic supplementary material

Supplemental Table 1

Supplemental Table 2

Supplemental Table 3

Supplemental Table 4

Supplemental Table 5

Supplemental Table 6

## References

[1] Stephen AG, Esposito D, Bagni RK, McCormick F (2014). Dragging ras back in the ring. Cancer Cell..

[2] Pylayeva-Gupta Y, Grabocka E, Bar-Sagi D (2011). RAS oncogenes: weaving a tumorigenic web. Nat. Rev. Cancer.

[3] Malumbres M, Barbacid M (2003). RAS oncogenes: the first 30 years. Nat. Rev. Cancer.

[4] Sun H, Tonks NK, Bar-Sagi D (1994). *I*nhibition of Ras-induced DNA synthesis by expression of the phosphatase MKP-1. Science.

[5] Jimenez C (1998). Identification and characterization of a new oncogene derived from the regulatory subunit of phosphoinositide 3-kinase. EMBO J..

[6] Khosravi-Far R (1996). Oncogenic Ras activation of Raf/mitogen-activated protein kinase-independent pathways is sufficient to cause tumorigenic transformation. Mol. Cell. Biol..

[7] Urano T, Emkey R, Feig LA (1996). Ral-GTPases mediate a distinct downstream signaling pathway from Ras that facilitates cellular transformation. EMBO J..

[8] White MA, Vale T, Camonis JH, Schaefer E, Wigler MH (1996). A role for the Ral guanine nucleotide dissociation stimulator in mediating Ras-induced transformation. J. Biol. Chem..

[9] Cowley S, Paterson H, Kemp P, Marshall CJ (1994). Activation of MAP kinase kinase is necessary and sufficient for PC12 differentiation and for transformation of NIH 3T3 cells. Cell.

[10] Cox AD, Der CJ (2003). The dark side of Ras: regulation of apoptosis. Oncogene.

[11] Ramji DP, Foka P (2002). CCAAT/enhancer-binding proteins: structure, function and regulation. Biochem. J..

[12] Tsukada Junichi, Yoshida Yasuhiro, Kominato Yoshihiko, Auron Philip E. (2011). The CCAAT/enhancer (C/EBP) family of basic-leucine zipper (bZIP) transcription factors is a multifaceted highly-regulated system for gene regulation. Cytokine.

[13] House JS, Zhu S, Ranjan R, Linder K, Smart RC (2010). C/EBPalpha and C/EBPbeta are required for Sebocyte differentiation and stratified squamous differentiation in adult mouse skin. PLoS ONE.

[14] Matsusaka T (1993). Transcription factors NF-IL6 and NF-kB synergistically activate transcription of the inflammatory cytokine interleukin 6 and interleukin 8. PNAS.

[15] Akagi T (2008). Impaired response to GM-CSF and G-CSF, and enhanced apoptosis in C/EBPbeta-deficient hematopoietic cells. Blood.

[16] Armstrong DA, Phelps LN, Vincenti MP (2009). CCAAT enhancer binding protein-beta regulates matrix metalloproteinase-1 expression in interleukin-1beta-stimulated A549 lung carcinoma cells. Mol. Cancer Res..

[17] Nakajima T (1993). Phosphorylaton at threonine-235 by a ras dependent mitogen-activated protein kinase cascade is essential for transcription factor NF-IL6. Proc. Natl Acad. Sci. USA.

[18] Zhu S, Yoon K, Sterneck E, Johnson PF, Smart RC (2002). CCAAT/enhancer binding protein-beta is a mediator of keratinocyte survival and skin tumorigenesis involving oncogenic Ras signaling. Proc. Natl Acad. Sci. USA.

[19] Buck M, Poli V, Geer Pvd, Chojkier M, Hunter T (1999). Phosphorylation of rat serine 105 or mouse threonine 217 in C/EBPbeta is required for hepatocyte proliferation induced by TGFalpha. Mol. Cell.

[20] Cho IJ, Woo NR, Kim SG (2008). The identification of C/EBPbeta as a transcription factor necessary for the induction of MAPK phosphatase-1 by toll-like receptor-4 ligand. Arch. Biochem. Biophys..

[21] Lu YC (2009). Differential role for c-Rel and C/EBPbeta/delta in TLR-mediated induction of proinflammatory cytokines. J. Immunol..

[22] Ewing SJ, Zhu S, Zhu F, House JS, Smart RC (2008). C/EBPbeta represses p53 to promote cell survival downstream of DNA damage independent of oncogenic Ras and p19(Arf). Cell Death Differ..

[23] Yoon K, Zhu S, Ewing SJ, Smart RC (2007). Decreased survival of C/EBP beta-deficient keratinocytes is due to aberrant regulation of p53 levels and function. Oncogene.

[24] Buck M, Poli V, Hunter T, Chojkier M (2001). C/EBPbeta phosphorylation by RSK creates a functional XEXD caspase inhibitory box critical for cell survival. Mol. Cell.

[25] Wessells J, Yakar S, Johnson PF (2004). Critical prosurvival roles for C/EBP beta and insulin-like growth factor I in macrophage tumor cells. Mol. Cell. Biol..

[26] Aguilar-Morante D., Cortes-Canteli M., Sanz-Sancristobal M., Santos A., Perez-Castillo A. (2011). Decreased CCAAT/enhancer binding protein β expression inhibits the growth of glioblastoma cells. Neuroscience.

[27] Anastasov N., Bonzheim I., Rudelius M., Klier M., Dau T., Angermeier D., Duyster J., Pittaluga S., Fend F., Raffeld M., Quintanilla-Martinez L. (2009). C/EBP  expression in ALK-positive anaplastic large cell lymphomas is required for cell proliferation and is induced by the STAT3 signaling pathway. Haematologica.

[28] Bundy LM, Sealy L (2003). CCAAT/enhancer binding protein beta (C/EBPbeta)-2 transforms normal mammary epithelial cells and induces epithelial to mesenchymal transition in culture. Oncogene.

[29] Duprez E (2004). A new role for C/EBPbeta in acute promyelocytic leukemia. Cell Cycle.

[30] Homma J (2006). Increased expression of CCAAT/enhancer binding protein beta correlates with prognosis in glioma patients. Oncol. Rep..

[31] Kim MH, Minton AZ, Agrawal V (2009). C/EBPbeta regulates metastatic gene expression and confers TNF-alpha resistance to prostate cancer cells. Prostate.

[32] Li W (2005). A gene expression signature for relapse of primary wilms tumors. Cancer Res..

[33] Pal R (2009). C/EBPbeta regulates transcription factors critical for proliferation and survival of multiple myeloma cells. Blood.

[34] Rask K (2000). Increased expression of the transcription factors CCAAT-rnhancer binding brotein-beta and C/EBP-zeta correlate with invasiveness of human colorectal cancer. Int. J. Cancer.

[35] Sundfeldt K (1999). The expression of CCAAT/enhancer binding protein in human ovary in vivo: specific increase in C/EBPbeta dering epithelial tumour progression. Br. J. Cancer.

[36] Piva R (2006). Functional validation of the anaplastic lymphoma kinase signature identifies CEBPB and BCL2A1 as critical target genes. J. Clin. Invest..

[37] Hanahan D, Weinberg RA (2011). Hallmarks of cancer: the next generation. Cell.

[38] Luo J, Solimini NL, Elledge SJ (2009). Principles of cancer therapy: oncogene and non-oncogene addiction. Cell.

[39] Downward J (2015). RAS synthetic lethal screens revisited: still seeking the elusive prize?. Clin. Cancer Res..

[40] Kaelin WG (2005). The concept of synthetic lethality in the context of anticancer therapy. Nat. Rev. Cancer.

[41] O'Neil Nigel J., Bailey Melanie L., Hieter Philip (2017). Synthetic lethality and cancer. Nature Reviews Genetics.

[42] Weinstein IB (1997). Disorders in cell circuitry associated with multistage carcinogenesis: exploitable targets for cancer prevention and therapy. Clin. Cancer Res..

[43] Vasioukhin V, Degenstein L, Wise B, Fuchs E (1999). The magical touch: genome targeting in epidermal stem cells induced by tamoxifen application to mouse skin. Proc. Natl Acad. Sci. USA.

[44] Sterneck E, Zhu S, Ramirez A, Jorcano JL, Smart RC (2006). Conditional ablation of C/EBP beta demonstrates its keratinocyte-specific requirement for cell survival and mouse skin tumorigenesis. Oncogene.

[45] Jonkers J (2001). Synergistic tumor suppressor activity of BRCA2 and p53 in a conditional mouse model for breast cancer. Nat. Genet..

[46] Schneider CA, Rasband WS, Eliceiri KW (2012). NIH Image to ImageJ: 25 years of image analysis. Nat. Methods.

[47] Kugelberg E (2005). Establishment of a superficial skin infection model in mice by using *Staphylococcus aureus* and *Streptococcus pyogenes*. Antimicrob. Agents Chemother..

[48] Dobin A (2013). STAR: ultrafast universal RNA-seq aligner. Bioinformatics.

[49] Love MI, Huber W, Anders S (2014). Moderated estimation of fold change and dispersion for RNA-seq data with DESeq2. Genome Biol..

[50] Benjamini, Y. and Hochberg, Y. Controlling the false discovery rate: a practical and powerful approach to multiple testing. *J. R. Stat. Soc. Ser. B (Methodol.).* **57**: 289–300 (1995).

[51] Subramanian A (2005). Gene set enrichment analysis: a knowledge-based approach for interpreting genome-wide expression profiles. Proc. Natl Acad. Sci. USA.

[52] Mootha VK (2003). PGC-1alpha-responsive genes involved in oxidative phosphorylation are coordinately downregulated in human diabetes. Nat. Genet..

[53] Abel EL, Angel JM, Kiguchi K, DiGiovanni J (2009). Multi-stage chemical carcinogenesis in mouse skin: fundamentals and applications. Nat. Protoc..

[54] Quintanilla M, Brown K, Ramsden M, Balmain A (1986). Carcinogen-specific mutation and amplification of Ha-ras during mouse skin carcinogenesis. Nature.

[55] Banin S (1998). Enhanced phosphorylation of p53 by ATM in response to DNA damage. Science.

[56] Canman CE (1998). Activation of the ATM kinase by ionizing radiation and phosphorylation of p53. Science.

[57] Appella E, Anderson CW (2001). Post-translational modifications and activation of p53 by genotoxic stresses. Eur. J. Biochem..

[58] Kuo LJ, Yang LX (2008). Gamma-H2AX - a novel biomarker for DNA double-strand breaks. In Vivo.

[59] Gaillard H, Garcia-Muse T, Aguilera A (2015). Replication stress and cancer. Nat. Rev. Cancer.

[60] Ferreira KS (2012). Caspase-3 feeds back on caspase-8, Bid and XIAP in type I Fas signaling in primary mouse hepatocytes. Apoptosis.

[61] Villunger A (2003). p53- and drug-induced apoptotic responses mediated by BH3-only proteins puma and noxa. Science.

[62] Puthalakath H (2007). ER stress triggers apoptosis by activating BH3-only protein Bim. Cell.

[63] Sano R, Reed JC (2013). ER stress-induced cell death mechanisms. Biochim. Biophys. Acta.

[64] Newton K, Strasser A (2000). Ionizing radiation and chemotherapeutic drugs induce apoptosis in lymphocytes in the absence of Fas or FADD/MORT1 signaling. Implications for cancer therapy. J. Exp. Med..

[65] Kuribayashi K (2008). TNFSF10 (TRAIL), a p53 target gene that mediates p53-dependent cell death. Cancer Biol. Ther..

[66] Finnberg N (2005). DR5 knockout mice are compromised in radiation-induced apoptosis. Mol. Cell. Biol..

[67] Wang S, El-Deiry WS (2003). TRAIL and apoptosis induction by TNF-family death receptors. Oncogene.

[68] Safa AR (2012). c-FLIP, a master anti-apoptotic regulator. Exp. Oncol..

[69] Honda K, Taniguchi T (2006). IRFs: master regulators of signalling by Toll-like receptors and cytosolic pattern-recognition receptors. Nat. Rev. Immunol..

[70] Miciak J, Bunz F (2016). Long story short: p53 mediates innate immunity. Biochim. Biophys. Acta.

[71] Munoz-Fontela C, Mandinova A, Aaronson SA, Lee SW (2016). Emerging roles of p53 and other tumour-suppressor genes in immune regulation. Nat. Rev. Immunol..

[72] Zhang F, Sriram S (2009). Identification and characterization of the interferon-beta-mediated p53 signal pathway in human peripheral blood mononuclear cells. Immunology.

[73] Zou B (2012). XIAP-associated factor 1 (XAF1), a novel target of p53, enhances p53-mediated apoptosis via post-translational modification. Mol. Carcinog..

[74] Munoz-Fontela C (2008). Transcriptional role of p53 in interferon-mediated antiviral immunity. J. Exp. Med..

[75] Ivashkiv LB, Donlin LT (2014). Regulation of type I interferon responses. Nat. Rev. Immunol..

[76] Townsend PA (2004). STAT-1 interacts with p53 to enhance DNA damage-induced apoptosis. J. Biol. Chem..

[77] Pampin M, Simonin Y, Blondel B, Percherancier Y, Chelbi-Alix MK (2006). Cross talk between PML and p53 during poliovirus infection: implications for antiviral defense. J. Virol..

[78] Micali OC (2007). Silencing of the XAF1 gene by promoter hypermethylation in cancer cells and reactivation to TRAIL-sensitization by IFN-beta. BMC Cancer.

